# RVG-modified exosomes derived from mesenchymal stem cells rescue memory deficits by regulating inflammatory responses in a mouse model of Alzheimer’s disease

**DOI:** 10.1186/s12979-019-0150-2

**Published:** 2019-05-13

**Authors:** Guo-hong Cui, Hai-dong Guo, Han Li, Yu Zhai, Zhang-bin Gong, Jing Wu, Jian-sheng Liu, You-rong Dong, Shuang-xing Hou, Jian-ren Liu

**Affiliations:** 1 Department of Neurology, Shanghai No. 9 People’s Hospital, Shanghai Jiaotong University School of Medicine, Shanghai, 200011 China; 2 Department of Anatomy, School of Basic Medicine, Shanghai University of Traditional Chinese Medicine, Shanghai, 201203 China; 3 Department of Biochemistry, School of Basic Medicine, Shanghai University of Traditional Chinese Medicine, Shanghai, 201203 China; 4 Department of Neurology, Shanghai Pudong Hospital, Fudan University Pudong Medical Center, Shanghai, 201399 China

**Keywords:** Alzheimer’s disease, Exosomes, Targeting, Mesenchymal stem cells, Inflammatory cytokine

## Abstract

**Background:**

Exosomes are lipid-bilayer enclosed nano-sized vesicles that transfer functional cellular proteins, mRNA and miRNAs. Mesenchymal stem cells (MSCs) derived exosomes have been demonstrated to prevent memory deficits in the animal model of Alzheimer’s disease (AD). However, the intravenously injected exosomes could be abundantly tracked in other organs except for the targeted regions in the brain. Here, we proposed the use of central nervous system-specific rabies viral glycoprotein (RVG) peptide to target intravenously-infused exosomes derived from MSCs (MSC-Exo) to the brain of transgenic APP/PS1 mice. MSC-Exo were conjugated with RVG through a DOPE-NHS linker.

**Results:**

RVG-tagged MSC-Exo exhibited improved targeting to the cortex and hippocampus after being administered intravenously. Compared with the group administered MSC-Exo, in the group administered RVG-conjugated MSC-Exo (MSC-RVG-Exo) plaque deposition and Aβ levels were sharply decreased and activation of astrocytes was obviously reduced. The brain targeted exosomes derived from MSCs was better than unmodified exosomes to improve cognitive function in APP/PS1 mice according to Morris water maze test. Additionally, although MSC-Exo injected intravenously reduced the expression of pro-inflammatory mediators TNF-α, IL-β, and IL-6, but the changes of anti-inflammatory factors IL-10 and IL-13 were not obvious. However, administration of MSC-RVG-Exo significantly reduced the levels of TNF-α, IL-β, and IL-6 while significantly raised the levels of IL-10, IL-4 and IL-13.

**Conclusions:**

Taken together, our results demonstrated a novel method for increasing delivery of exosomes for treatment of AD. By targeting exosomes to the cortex and hippocampus of AD mouse, there was a significant improvement in learning and memory capabilities with reduced plaque deposition and Aβ levels, and normalized levels of inflammatory cytokines.

**Electronic supplementary material:**

The online version of this article (10.1186/s12979-019-0150-2) contains supplementary material, which is available to authorized users.

## Introduction

Alzheimer’s disease (AD), the most prevalent type of dementia, is characterized by excessive deposition of amyloid-β peptide (Aβ) and neurofibrillary tangles, which cause neuronal dysfunction and loss ultimately [1]. Rapid advances in stem-cell biology have provided a promising novel therapy for AD. A huge volume of data has demonstrated that stem cell transplantation improved cognition, learning, and memory by replacement of damaged neurons, release of neurotrophic factors, and clearance of Aβ plaques [2]. Our previous study showed that transplantation of neural stem cells (NSCs) improved cognitive function in an Aβ-infused model of AD via neuron differentiation and paracrine action [3]. In addition, chronic inflammation is involved in the pathogenesis of AD, and mesenchymal stem cells (MSCs) appear to exert anti-inflammatory effects by modulating the balance of proinflammatory and anti-inflammatory factors [4].

Recent data showed that stem cell-derived exosomes might be a new strategy for several central nervous system (CNS) disorders. The exosomes are small biological lipid membrane vesicles with diameters ranging from 30~100 nm found in various cell types and the major mediators of intercellular communication through shuttling biologically active molecules to targeted recipient cells [5]. The transfer of exosomal cargo provides a rationale for the many reported stem cell-based therapeutic outcomes. For example, MSC-derived exosomes benefit neurite remodeling and functional recovery after stroke by mediating the miR-133b transfer to astrocytes and neurons [6]. Moreover, it has been reported that human adipose MSC-derived exosomes contain functional neprilysin, a major Aβ-degrading enzyme and, thus, have the potential to reduce the pathological accumulation of Aβ in AD [7]. In our previous study, the expression of miR-21 in MSC-derived exosomes was involved in modulation of the inflammatory response and improvement of cognitive function in an APP/PS1 double transgenic model of AD [8]. In addition, miRNA-181c expression in MSC-exosomes reduced burn-induced inflammation by down-regulating the toll-like receptor 4 signaling pathway [9].

Although the exosomes have the ability to cross the blood-brain barrier (BBB), a recent dynamic biodistribution study of exosomes revealed a significant amount of exosomes accumulated in the spleen and liver, and very limited exosomes signals were detected in the brain after systemic administration [10]. Thus, we propose that surface modified stem cell-derived exosomes targeted to the brain are agents with superior therapeutic potential to prevent memory deficits in AD. The CNS-specific rabies viral glycoprotein (RVG) peptide interacts specifically with the acetylcholine receptor to enable viral entry into neuronal cells [11]. Lydia et al. acquired targeted exosomes by engineering the exosomes to express RVG peptide and intravenous injection of these engineered exosomes delivered siRNA specifically to the mouse brain [12]. Furthermore, the targeted RVG exosomes loaded with opioid receptor Mu (MOR) siRNA was a potential novel strategy for the treatment of morphine relapse by down-regulating MOR expression levels [13].

In this study, we created brain-targeting exosomes derived from MSCs, through the use of peptide RVG, to increase the efficacy of intravenously delivered exosomes. Then the RVG modified exosomes were intravenously administrated to APP/PS1 transgenic mice through the tail vein to observe its therapeutic effect through Morris water maze test. Plaque deposition and Aβ accumulation were detected through Thioflavin-S staining and ELISA, respectively. Additionally, the expression of pro-inflammatory and anti-inflammatory cytokines were determined through quantitative real-time polymerase chain reaction (qRT-PCR) and ELISA. This study presented a feasible way to develop exosomes targeted to the brain for treating AD.

## Materials and methods

### Animals

APP/PS1 double transgenic mice, B6C3-Tg (APPswe, PSEN1dE9) 85Dbo/J (original species No. 004462), were obtained from Jackson Laboratories (Bar Harbor, ME). The transgenic mice were maintained on a standard 12-h light/dark cycle at a constant temperature, with free access to food and water. This study was approved by the animal ethics committee of Shanghai University of TCM and the Animal Research Committee of Shanghai. All of the protocols were based on the “Guide for the Care and Use of Laboratory Animals” of the National Institutes of Health (USA) and efforts were made to minimize the number of animals used and any discomfort experienced.

### Isolation and culture of MSCs

To isolate bone marrow, mice were killed by cervical dislocation and the hind limbs were dissected from the trunk of the body with care not to damage the femur. Bisect each hind limb by cutting through the knee joint. Remove the muscle and connective tissue from both the tibia and the femur. Cut the ends of the tibia and femur just below the end of the marrow cavity and flush the marrow using a 27-gauge needle attached to a 10-ml syringe containing Dulbecco’s Modified Eagle’s medium (DMEM, Gibco, San Diego, USA). The medium with bone marrow was centrifuged and the cell pellet was resuspended in DMEM supplemented with 15% fetal bovine serum (FBS, Gibco) and then cultured at 37 °C under a 5% CO_2_ atmosphere. The medium was changed at 48 h. For subculture, cells were detached with 0.25% trypsin-ethylene diamine tetra-acetic acid (Gibco Invitrogen, USA) and seeded in DMEM with 15% FBS [14]. The characterization of cell surface markers (CD29, CD44, CD45) were detected through immunofluorescence staining. The cells were incubated with the primary antibodies (1:100, Abcam, USA) overnight at 4 °C after being fixed with 4% paraformaldehyde. Then the cells were labeled with Alexa Flour 488 secondary antibody (1:200, Invitrogen) for 2 h at room temperature.

### Isolation and injection of exosomes

FBS was spun at 100,000 g for 2 h to avoid contamination by the FBS-derived exosomes before the experiment. The cell supernatant was collected and exosomes were isolated using gradient centrifugation as previously described [15]. In brief, the cell debris and large membrane vesicles were removed by sequential centrifugation at 300 g for 10 min, 2000 g for 10 min, and 10,000 g for 30 min, followed by filtration using 0.22 μm syringe filters. Then, the cleared supernatant was transferred to a fresh tube and spun at 100,000 g for 70 min. After that, the supernatant was removed completely and the pellet was washed with phosphate buffer solution (PBS) to collect the exosomes. The sizes and concentrations of exosomes were determined by nanoparticle tracking analysis (NTA; NanoSight, Malvern, Worcestershire, United Kingdom).

### Exosome tagging

The exosomes were tagged with RVG peptide (YTIWMPENPRPGTPCDIFTNSRGKRASNG) shown to target the CNS. The peptide was custom synthesized and then was conjugated to the exosomes using modifications of previously described methods [16]. DOPE-NHS (dioleoylphosphatidylethanolamine N-hydroxysuccinimide; COATSOME® FE-8181SU5, NOF America; White Plains, NY) and RVG peptide were combined and allowed to react for 1 h to obtain the DOPE-RVG. The DOPE-RVG was then incubated with the exosomes derived from MSCs. The DOPE-RVG to exosome labelling ratio was determined as 500:1 considering the length of peptide and radius of exosome. The modified exosomes subsequently were labelled with lipophilic dye DiI (1,1′-Dioctadecyl-3,3,3′,3′-Tetramethylindocarbocyanine Perchlorate, ThermoFisher Scientific, Waltham, MA) for the biodistribution detection.

### Injection of exosomes

The 7-month-old APP/PS1 mice were intravenously treated with PBS (AD), exosomes derived from MSCs (MSC-Exo) or RVG-conjugated MSC-Exo (MSC-RVG-Exo) monthly for 4 months (13 mice for each group). In all three groups, the injection volume was 100 μl. In both the MSC-Exo and MSC-RVG-Exo treated groups, the treatments were 5 × 10^11^ MSC-Exo or MSC-RVG-Exo in 100 μl PBS, respectively. There was no drop-out during normal breading and the injection period. To detect the presence of the exosomes in the brain, 3 mice in each group were euthanized at 5 h after injection and the brain slides were examined by confocal microscopy after counterstained with 4′,6-diamidino-2-phenylindole (DAPI).

### Behavior test

The Morris water maze test was performed to ascertain the spatial memory performance after exosome administration. The water maze test includes spatial training and probe test. Briefly, the mice were placed into a black-painted circular water tank (1.0 m diameter, 30 cm height) which contains a platform in the center of North-West quadrant (target quadrant). The platform was located about 1 cm beneath the water surface and different geometric shapes were positioned around the tank to provide spatial orientation. The mice were given 60 s to find this invisible platform in the first 5 days for spatial acquisition tests. Each mouse was placed in the water maze randomly from one of four different quadrants. The time to find the platform was recorded as the escape latency. Mice that did not find the platform within 60 s were manually guided to the platform by the experimenter. The animals underwent 4 trials per day and the swim traces were recorded by a video camera that was linked to a computer. In the post-training probe trial test, the platform was removed after training for 5 days. The mouse was placed in the opposite quadrant of the target quadrant in the maze, and allowed to swim for 60 s to assess learning and memory capabilities. The number of platform location crossings and time spent in the target quadrant were measured during this period.

### Tissue preparation and Thioflavin-S staining

The mice were anesthetized via 1% pentobarbital sodium (45 mg/kg, i.p.) after the behavior test and transcardially perfused with a 0.9% saline solution followed by 4% paraformaldehyde via the left ventricle. The brain tissue samples which cover the complete hippocampus were fixed with 4% paraformaldehyde at 4 °C overnight and embedded in Optimal Cutting Temperature (OCT; Sakura Finetek USA, Inc.) compound after osmotic dehydration in sucrose solutions. Sequential 12 μm coronal sections were cut on a cryostat and stored at − 20 °C until staining. The sections were immersed in 0.5% Thioflavin-S (Sigma-Aldrich) dissolved in 50% ethanol for 8 min. Subsequently, the sections were washed three times with 50% ethanol and then twice in distilled water. The slides were mounted and images were acquired with a fluorescence microscope (IX53, Olympus). Five fields in the cortex and hippocampus of the mice were selected randomly and pixel areas of amyloid plaques were quantified using ImageJ software (NIH, Bethesda, MD, USA).

### Immunofluorescence staining

After being permeabilized with 0.1% Triton X-100 and blocked with 5% normal goat serum for 1 h at room temperature, the slides were incubated with rabbit anti-GFAP (1:200, Abcam, USA) overnight at 4 °C. Corresponding secondary antibody as Alexa Fluor 488 (1:200, Invitrogen) was incubated for 2 h at room temperature. Five fields in each mouse were selected randomly and the fluorescence intensity was analyzed using Image-Pro Plus. The expression of GFAP was normalized to the values of AD group.

To examine the biodistribution of DiI-labeled exosomes in mouse brain, the brain slides were stained with DAPI, and images were taken using an immunofluorescence microscope. The mean fluorescence intensity was analyzed using Image-Pro Plus and the engraftment of DiI-labeled exosomes was normalized to the values of MSC-Exo group.

### Enzyme linked immunosorbent assay (ELISA)

The concentration of soluble and insoluble Aβ_40_ and Aβ_42_ in the brain was detected with ELISA analysis following the manufacturer’s instructions of specific ELISA kits (Invitrogen, Camarillo, CA). The hippocampus and frontal cortex from one hemisphere were homogenized in ice-cold protein extraction buffer containing a protease inhibitor and centrifuged at 4 °C for 30 min to obtain the supernatants as the soluble extract. The protein concentration was quantified using a BCA protein assay (Thermo Fisher Scientific, Waltham, MA) for sample normalization. Detergent-insoluble Aβ_40_ and Aβ_42_ was detected by extracting pellets in 5 M guanidine HCl buffer, followed by a 1:20 dilution in lysis buffer. Each experimental sample was run in duplicate, and the concentrations of Aβ in sample were determined using the standard curve. Values are expressed as a relative % of the AD group. The concentrations of pro-inflammatory or anti-inflammatory cytokines in the brain were also detected through ELISA (RayBiotech, GA, USA) according to the manufacturer’s instructions.

### qRT-PCR

Quantification of pro-inflammatory or anti-inflammatory gene expression was performed by qRT-PCR. The hippocampus and frontal cortex from one hemisphere were extracted and homogenized on ice immediately. Total RNA was extracted from the samples using TRIzol reagent (Invitrogen) according to the manufacturer’s instructions. Subsequently, RNA samples (0.8 μg) were reverse transcribed using QuantiTect Reverse Transcription kit (Qiagen). Real-time PCR of cDNA was performed on ABI 7500 Real-Time PCR system (Applied Biosystems) using SYBR Green ready mix (Applied Biosystems). All reactions were done in triplicate. The relative expression levels were calculated according to the comparative CT method (2^−ΔΔCt^).

## Statistical analysis

All graphs are represented as the mean ± standard deviation. The statistical significance was evaluated using Student’s t-test when comparing with two groups. Multiple groups were compared using one-way ANOVA followed by least significant difference tests. Results were considered statistically significant when *P* < 0.05. All statistical analysis was performed using SPSS 23.0 (SPSS Inc., USA).

## Results

### Engraftment of targeted exosomes derived from MSCs in the brain

**Fig. 1:**
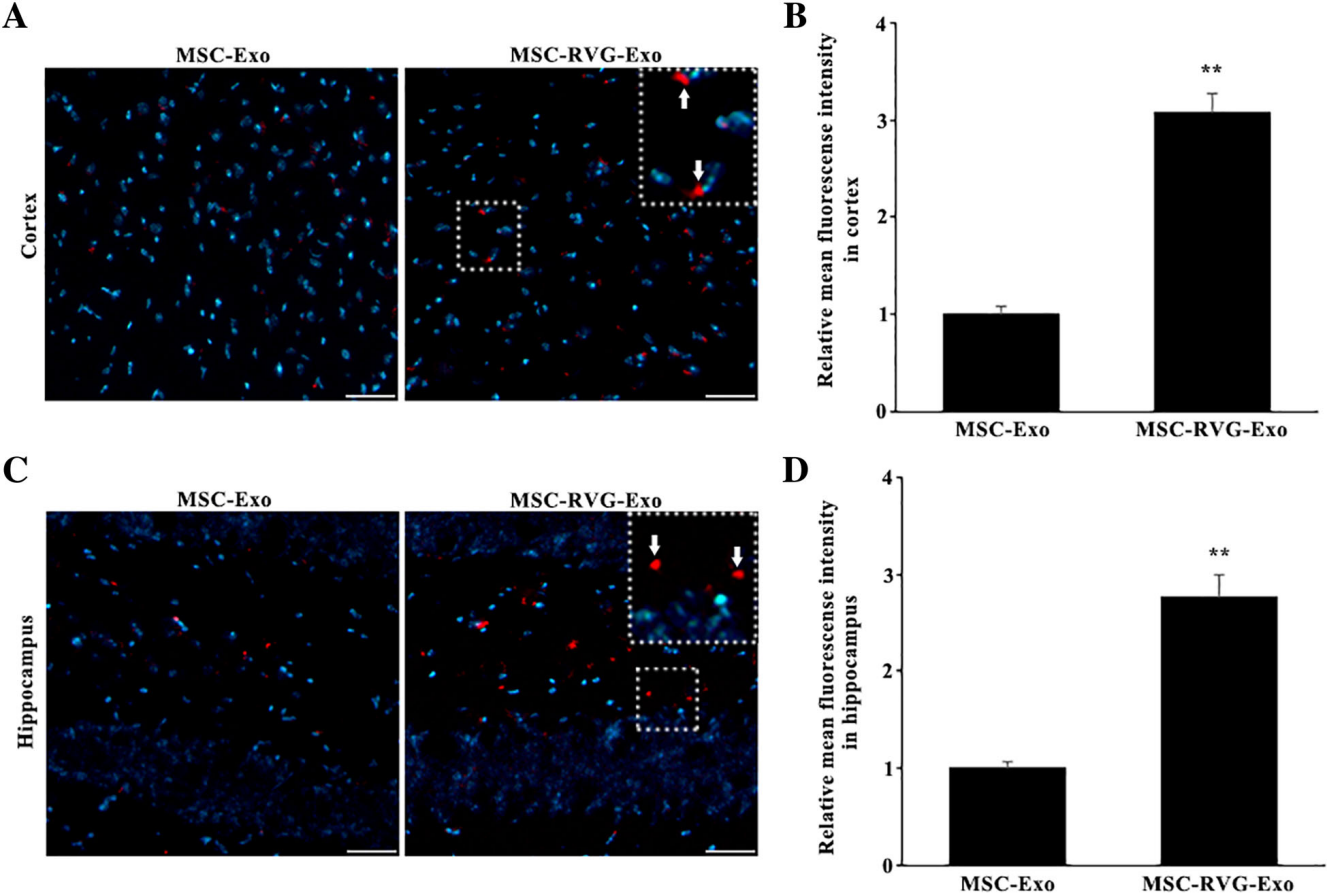
Enhanced engraftment of targeted exosomes derived from MSCs in the brain. The DiI-labeled exosomes were red and nuclei were counterstained with DAPI. The injected exosomes were detected in the cortex (**a**) and hippocampus (**c**) in both MSC-RVG-Exo group and MSC-Exo group. The number of injected exosomes was further compared by relative mean fluorescence intensity (**b**, **d**). **P < 0.01 versus the MSC-Exo group, by Student’s t-test; *n* = 3

The MSCs we harvested expressed CD29 and CD44 and were negative for CD45 (Additional file 1: Figure S1), which indicated that the purity of MSCs used in our study was high. To detect the presence of the exosomes derived from MSCs in the brain and whether RVG modification enhanced the engraftment of exosomes in the cortex and hippocampus, the slides of brain were observed under fluorescence microscope at 5 h after injection. The DiI-labeled exosomes were found in the cortex and hippocampus in both MSC-RVG-Exo group and MSC-Exo group. The number of injected exosomes was further compared by relative mean fluorescence intensity. Indeed, there was much more DiI-labeled exosomes in the cortex and hippocampus of the mice in the MSC-RVG-Exo group than that in the MSC-Exo group (Fig. 1a-d).

### Targeted exosomes derived from MSCs alleviated plaque deposition and Aβ accumulation

**Fig. 2:**
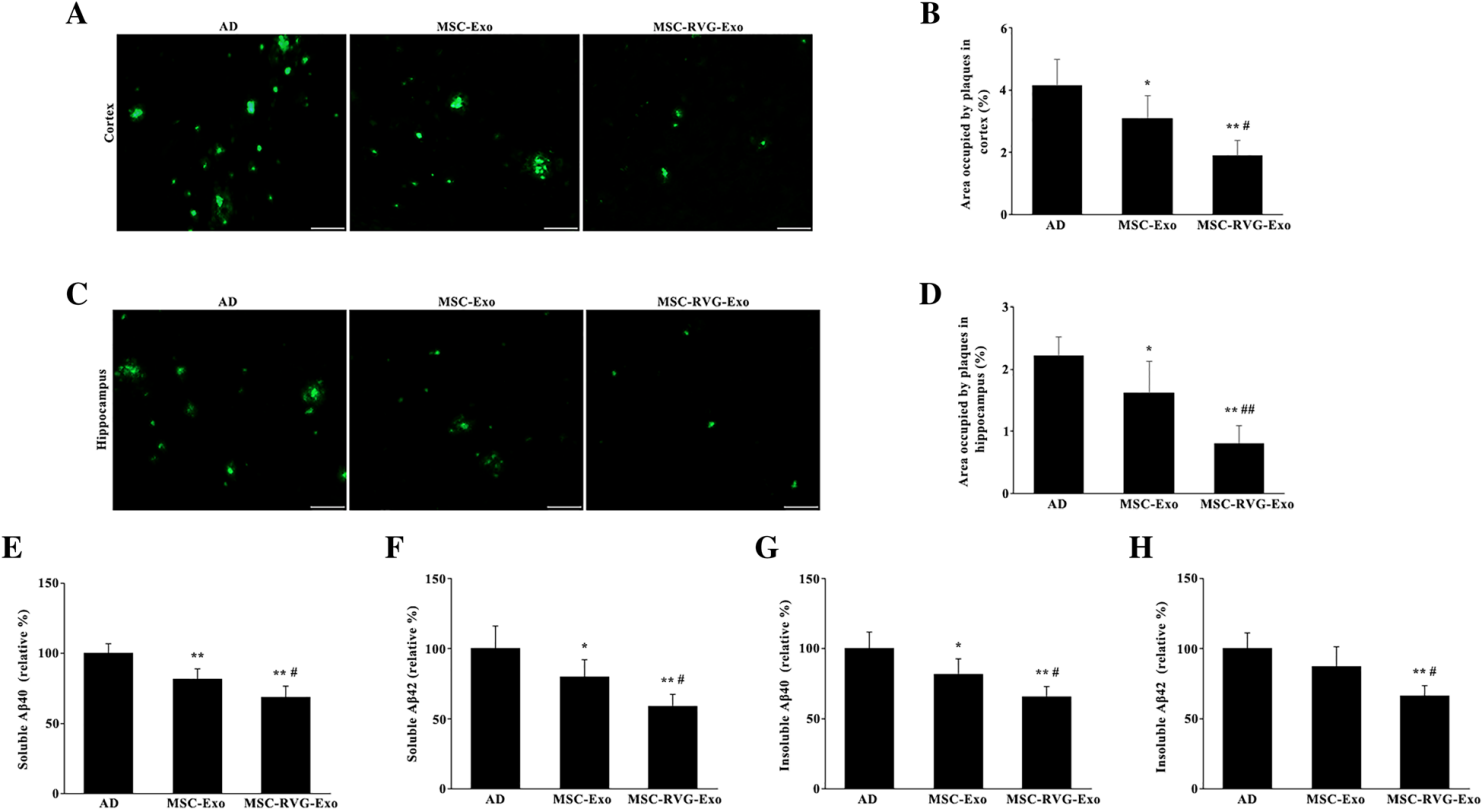
Targeted exosomes derived from MSCs reduced plaques deposition and Aβ accumulation in the brain. Thioflavin-S staining was performed to detect the plaques in the cortex (**a**, Scale bar = 50 μm) and quantitative analysis of the area occupied by plaques in the cortex (**b**) of APP/PS1 mice. The plaques deposition was also detected through Thioflavin-S staining in the hippocampus (**c**, Scale bar = 50 μm) and the area occupied by plaques in the hippocampus (**d**) was quantified. The plaque deposition area in the cortex and hippocampus of MSC-RVG-Exo treated mice were much lower than that in mice injected with MSC-Exo. The amount of soluble Aβ40 (**e**) and Aβ42 (**f**), and insoluble Aβ40 (**g**) and Aβ42 (**h**) in the brains were determined through a commercial ELISA kit. *P < 0.05 and **P < 0.01 versus the AD group. #*P* < 0.05 and ##*P* < 0.01 versus the MSC-Exo group, by one-way ANOVA; *n* = 5

To investigate the effect of MSC-RVG-Exo on amyloid plaque loading in the brain, two experiments, including Thioflavin-S staining and ELISA were performed. MSC-Exo treatment decreased plaque deposition in the cortex according to Thioflavin-S staining and interestingly there was a dramatic reduction in plaque deposition in the cortex of mice treated with MSC-RVG-Exo compared with MSC-Exo injected mice (Fig. 2a, b). Similarly, plaque deposition in the hippocampus was significantly reduced after treated by MSC-Exo or MSC-RVG-Exo. Furthermore, the plaque deposition area in the hippocampus of MSC-RVG-Exo treated mice was much lower than that in mice injected with MSC-Exo (Fig. 2c, d). To further investigate whether MSC-RVG-Exo injection contributed to Aβ clearance, the Aβ content in the brain was examined through an Aβ ELISA kit. The data showed that treatment with MSC-Exo reduced both soluble Aβ_40_ and soluble Aβ_42_ in the brains of APP/PS1 mice. Compared with the group of mice treated by MSC-Exo, the concentration of soluble Aβ_40_ and soluble Aβ_42_ was much lower in MSC-RVG-Exo treated mice (Fig. 2e, f). Additionally, the expression of insoluble Aβ_40_ in the group of MSC-Exo was much lower than that in the group of saline-injected AD mice. No significant decrease in the expression of insoluble Aβ_42_ was detected between the group treated with MSC-Exo and the group treated with saline. However, MSC-RVG-Exo treatment showed the lowest concentration of insoluble Aβ_40_ and insoluble Aβ_42_ in the brain and there was a significant difference between group MSC-RVG-Exo and MSC-Exo (Fig. 2g, h).

### Targeted exosomes derived from MSCs inhibited activation of astrocytes

**Fig. 3:**
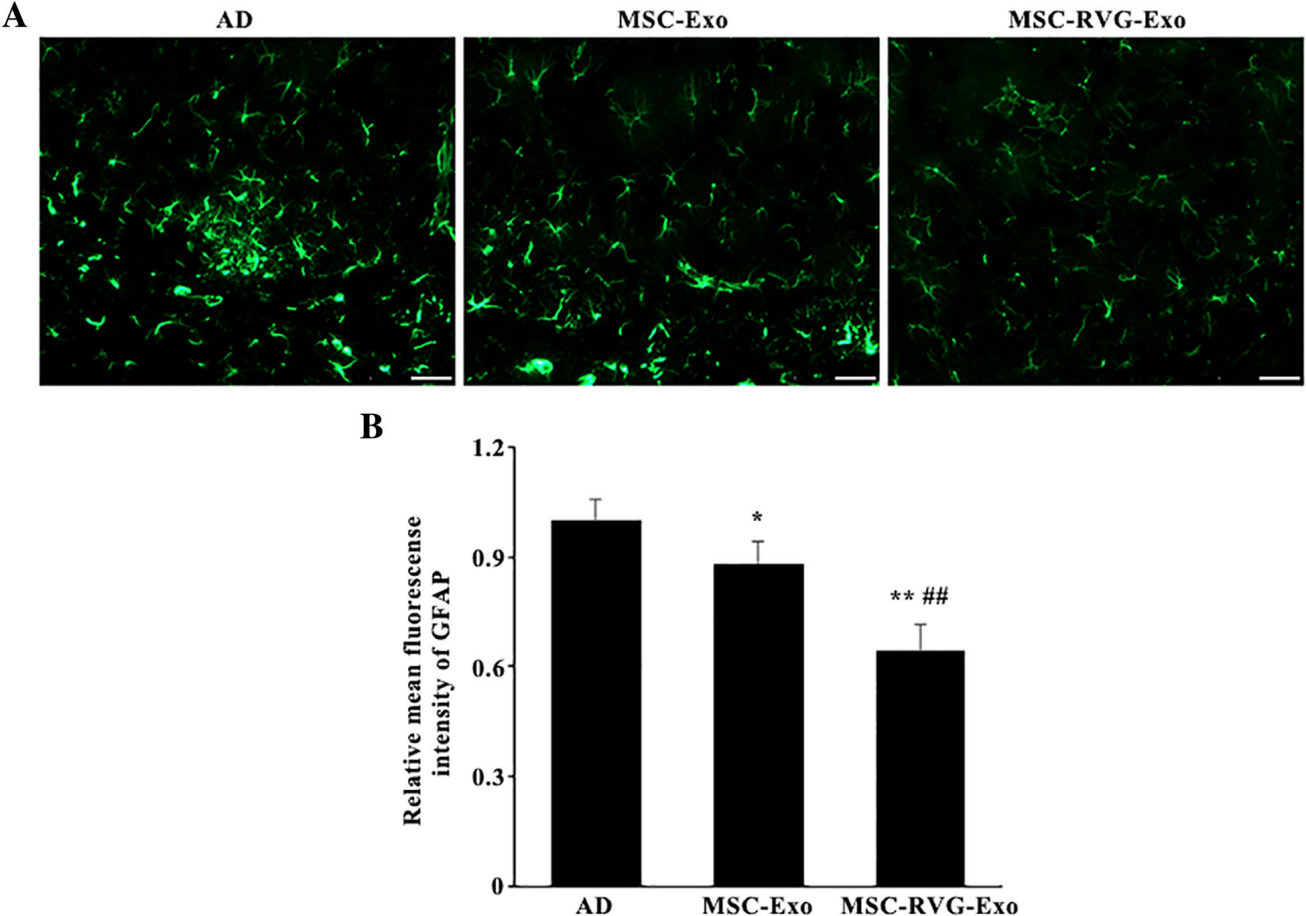
Targeted exosomes derived from MSCs repressed activation of astrocytes after injection intravenously. (**a**) Representative immunofluorescence images of GFAP protein expression in each group. (**b**) Quantification of the mean density of GFAP. The AD mice treated with MSC-RVG-Exo showed the lowest level of GFAP expression among the groups. Scale bar = 25 μm. *P < 0.05 and **P < 0.01 versus the AD group. ##*P* < 0.01 versus the MSC-Exo group, by one-way ANOVA; *n* = 5

Next, the activation of astrocytes was analyzed according to immunofluorescence staining. As shown in Fig. 3a, the expression of GFAP, one marker of astrocytes, was highly robust in AD mice injected with saline. Injection of MSC-Exo and MSC-RVG-Exo could significantly attenuate the expression of GFAP in the brain. GFAP is just not an astrocyte marker, but also the marker of astrocyte activation because of the morphologic changes of astrocyte could be observed after immunostaining of GFAP [17–19]. The processes of astrocytes in AD group become thicker and the number of processes was increased when visualized with antibodies against GFAP. However, there were more astrocytes in the groups of exosome injection, especially the group of MSC-RVG-Exo, showed their original tiled domains. Statistically, the AD mice treated with MSC-RVG-Exo showed the lowest expression of GFAP among the groups (Fig. 3b).

### Targeted exosomes derived from MSCs ameliorated spatial learning and memory impairments in APP/PS1 mice

**Fig. 4:**
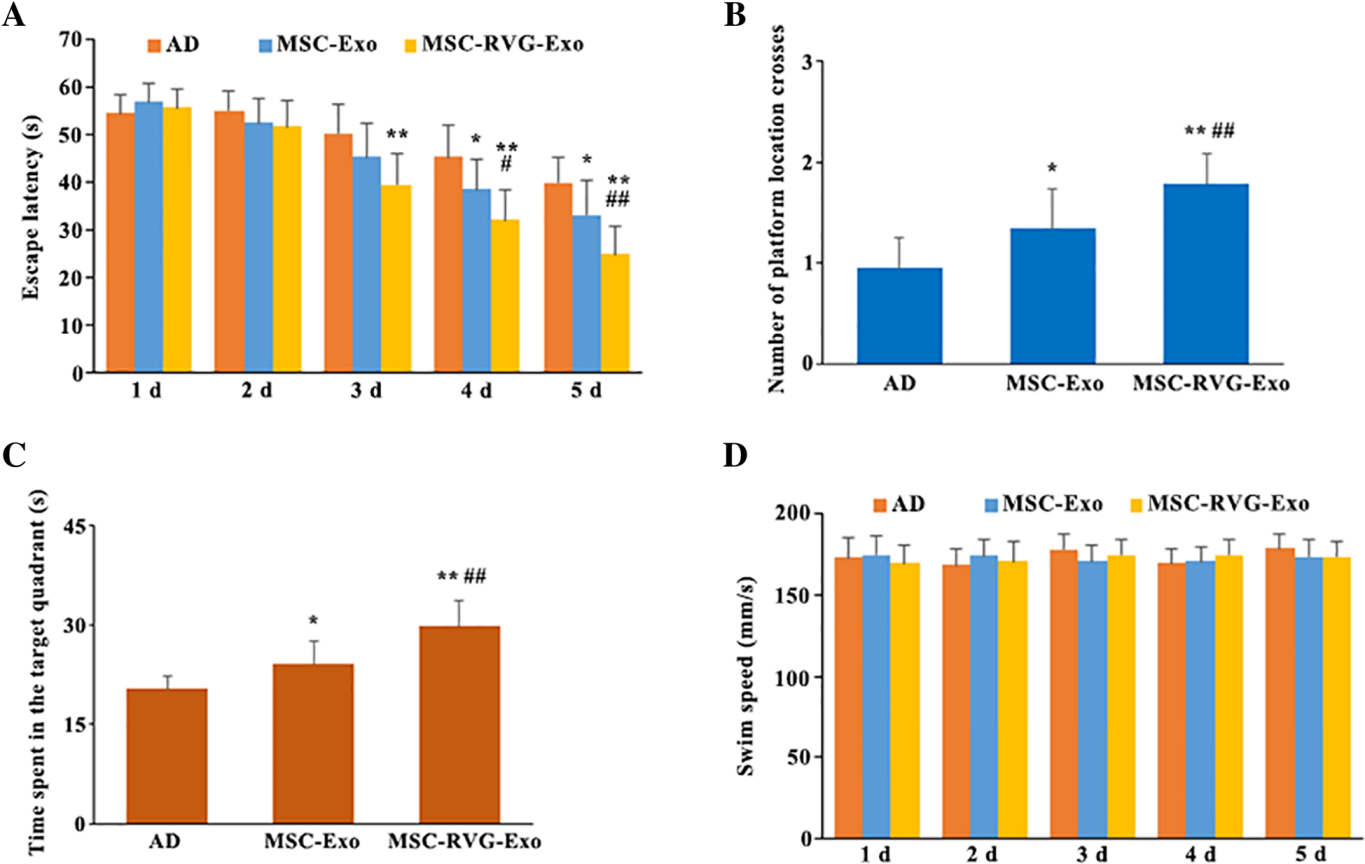
Targeted exosomes derived from MSCs improved spatial learning and memory performances in APP/PS1 mice. (**a**) MSC-RVG-Exo and MSC-Exo injection ameliorated spatial learning in AD mice as shown by mean escape latency during the acquisition training phase through Morris water maze test. The number of platform location crosses (**b**) and time spent in the target quadrant (**c**) were evaluated by post-training probe trials. Compared with the mice injected by MSC-Exo, the mice in the group of MSC-RVG-Exo showed increased number of platform location crossings and time spent in the target quadrant. (**d**) The swimming speeds were similar among the groups, which suggested that the improved ability of learning and memory in mice treated by MSC-RVG-Exo resulted from cognitive processes but not non-cognitive behavioral components. AD indicated the APP/PS1 mice injected with PBS, MSC-Exo indicated the APP/PS1 mice injected with exosomes derived from MSCs, MSC-RVG-Exo indicated the APP/PS1 mice injected with RVG-conjugated MSC-Exo. *P < 0.05 and **P < 0.01 versus the AD group. #*P* < 0.05 and ##*P* < 0.01 versus the MSC-Exo group, by one-way ANOVA; *n* = 10

Morris water maze test was performed to assess whether intravenous injection of brain targeted exosomes derived from MSCs could improve spatial learning and memory in APP/PS1 mice. As shown by mean escape latency during the acquisition training phase, spatial learning and memory was dramatically impaired in the saline-injected AD mice. MSC-Exo treatment significantly reduced the escape latency compared with saline-injected AD mice on days 4 and 5. From 3 days after acquisition training, mice treated with MSC-RVG-Exo presented obvious improved ability of learning and memory. Interestingly, mice injected with MSC-RVG-Exo showed much lower escape latency than that in the mice treated by MSC-Exo on days 4 and 5 (Fig. 4a). The number of platform location crosses was higher and the time spent in the target quadrant was longer for MSC-Exo than saline-injected AD mice. Compared with the mice treated by MSC-Exo, injection of MSC-RVG-Exo increased the number of platform location crossings and time spent in the target quadrant (Fig. 4b, c). The swimming speeds were similar among the groups, which suggested that the improved ability of learning and memory in mice treated by MSC-RVG-Exo resulted from cognitive processes but not non-cognitive behavioral components (Fig. 4d). Thus, the brain targeted exosomes derived from MSCs was better than unmodified exosomes to improve cognitive function in APP/PS1 mice.

### Targeted exosomes derived from MSCs decreased pro-inflammatory factors and increased anti-inflammatory factors

**Fig. 5:**
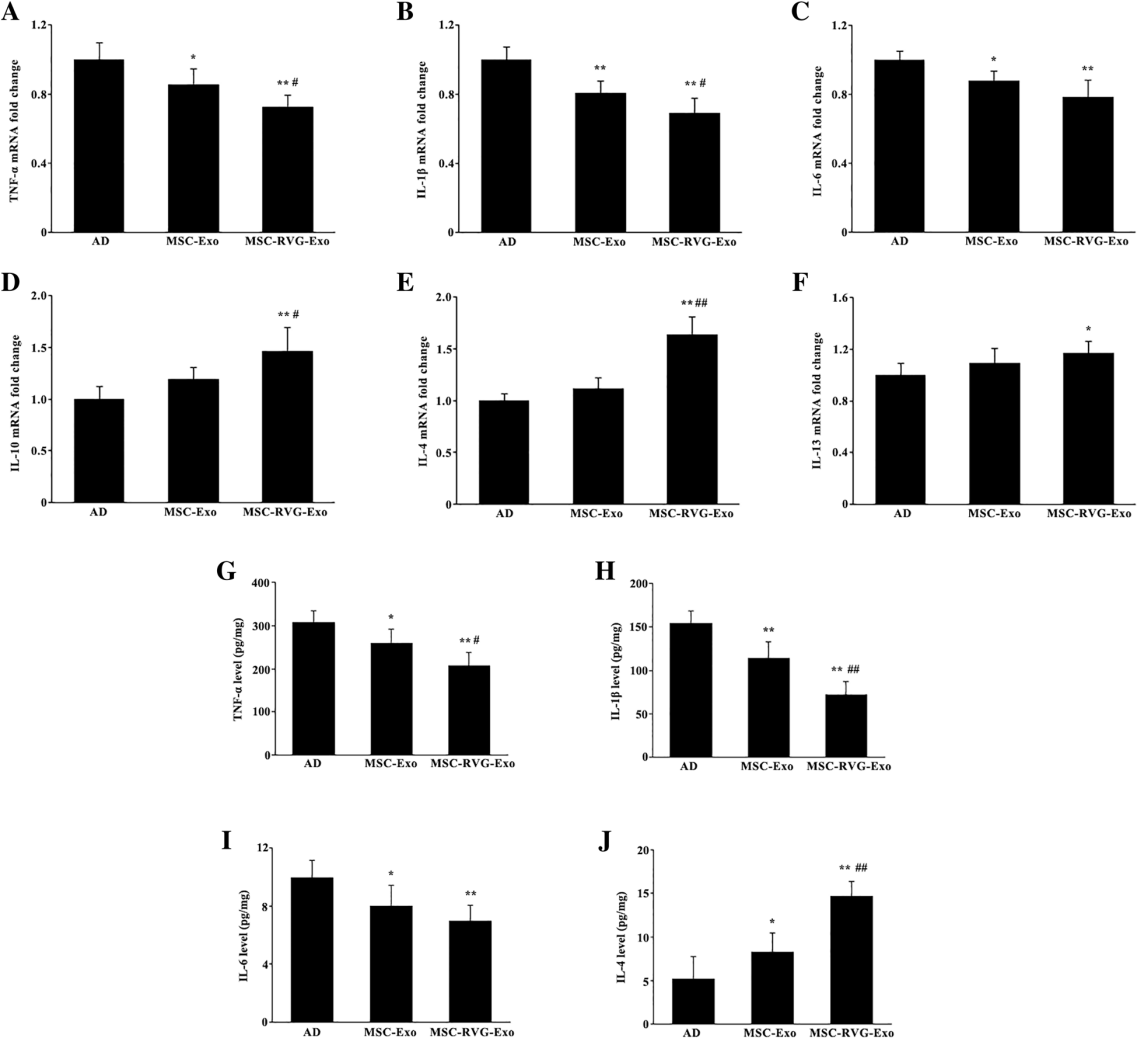
Targeted exosomes derived from MSCs regulated the expression of pro-inflammatory and anti-inflammatory cytokines. The mRNA expression of pro-inflammatory and anti-inflammatory cytokines was detected by qRT-PCR. The pro-inflammatory mediators TNF-α, IL-β, and IL-6 were significantly downregulated in the groups of MSC-Exo and MSC-RVG-Exo. The levels of TNF-α and IL-1β were much lower in the group of MSC-RVG-Exo than those in the group of MSC-Exo (**a**-**c**). After being treated by MSC-RVG-Exo, the mice showed elevated expression of anti-inflammatory cytokines IL-10, IL-4 and IL-13 (**d**-**f**). The concentrations of TNF-α, IL-β, IL-6 and IL-4 in the brain were further detected through ELISA and the data showed that the concentrations of these cytokines was similar to the expression patterns of mRNA level (**g**-**j**). *P < 0.05 and **P < 0.01 versus the AD group. #*P* < 0.05 and ##*P* < 0.01 versus the MSC-Exo group, by one-way ANOVA; *n* = 5

The neuroinflammatory responses play a major role in the development of AD pathologies, including the formation of neurotoxic amyloid plaque and cognitive deficits. The mRNA expression of pro-inflammatory and anti-inflammatory cytokines was determined through qRT-PCR. We found that that the pro-inflammatory mediators TNF-α, IL-β, and IL-6 were significantly downregulated in AD mouse brains after MSC-Exo treatment or MSC-RVG-Exo treatment. Compared with the group of MSC-Exo, the levels of TNF-α and IL-1β were much lower in the group treated with MSC-RVG-Exo (Fig. 5a-c). The expression of anti-inflammatory cytokines IL-10, IL-4 and IL-13 in the brain was only slightly upregulated after treated by MSC-Exo. However, MSC-RVG-Exo treatment significantly raised the level of IL-10, IL-4 and IL-13. Furthermore, the expression of IL-10 and IL-4 was significantly increased in MSC-RVG-Exo injected AD mice, compared with MSC-Exo injected AD mice (Fig. 5d-f). Moreover, the concentrations of TNF-α, IL-β, IL-6 and IL-4 determined by ELISA was similar to the expression patterns of mRNA level (Fig. 5g-j). Thus it appears that MSC-RVG-Exo treatment could act as an important regulator of both the pro-inflammatory and anti-inflammatory responses in the AD brain.

## Discussion

Currently, there are no treatments that halt or reverse the course of AD, and only palliative therapies exist. In our previous study, we reported that the APP/PS1mice treated with exosomes from MSCs showed obviously improved learning and memory [8]. However, the intravenously injected exosomes could be obviously tracked in lungs and spleen. Thus, it is interesting to investigate the enhancement of learning and memory performance in AD mice by targeting exosomes to the brain. The present study firstly demonstrated an effective method for increasing the potency of MSC-Exo for the treatment of AD through modification with targeted peptide RVG. MSC-RVG-Exo administration significantly alleviated memory deficits and effectively decreased Aβ levels and amyloid plaque loading in the brain of APP/PS1 mice. Overall, successful development of brain targeted exosomes may lead to a viable therapy for patients with AD.

The viral entry into neuronal cells through the interaction between RVG and nicotinic acetylcholine receptor (AchR) on neuronal cells [20, 21]. It has been confirmed that the potential of RVG to mediate transvascular delivery of small interfering RNA (siRNA) to the CNS [22]. Recently, RVG has been engineered to exosomal surface via fused protein lysosome-associated membrane glycoprotein 2b (Lamp2b)-RVG in order to achieve neuron-specific targeting [12]. Furthermore, the modified exosomes, with RVG fused to exosomal protein Lamp2b, could efficiently deliver miR-124 to the infarct site after ischemia [23]. In our study, DiI fluorescence ferried by exosomes distributed in the brain, which was consistent with the work reported by Haney in 2015 [15]. However, compared with unmodified exosomes, the concentrated fluorescence in the cortex and hippocampus showed high targeting efficiency of MSC-RVG-Exo. It was reported that receptor-mediated transcytosis by means of the α7 subunit of the AchR was probably involved in the process of exosome invasion in the brain after intravenous injection. The α7 subtype, is highly expressed in brain regions relevant to cognitive and memory functions and involved in the pathophysiologic processes underlying AD [24]. However, the expressions of AChRs were significantly decreased in AD patients according to biochemical analyses of postmortem brains [25]. There was evidence that extended exposure times and high concentrations of Aβ42 lead to dysregulation of α7 AChR, ERK MAPK, and CREB, as well as impaired memory and learning [26]. Besides, the significant decrease in α7 AChR protein expression takes place in the temporal cortex of AD brains. Thus, it’s worthwhile to overcome this issue to optimize exosomal therapy for targeting the CNS in the future.

The use of DOPE-NHS allows for peptides to be conjugated to exosomes and possibly other membrane-based nanoparticles. In this study, MSC derived exosomes were conjugated with RVG through a DOPE-NHS linker. After being administered intravenously, RVG-tagged MSC-Exo exhibited improved targeting to the cortex and hippocampus. The mice treated by MSC-RVG-Exo showed alleviated spatial learning and memory impairments remarkably. Cardiac stem cell-derived exosomes were also conjugated with cardiac homing peptide (CHP) through this method to target intravenously-infused exosomes to the infarcted heart. The CHP exosomes reduced fibrosis and scar size, and increased cellular proliferation and angiogenesis after myocardial infarction [27]. Except the RVG system, other moieties including peptides, ligands or receptor-specific antibodies has also been utilized to achieve targeting delivery to the brain [28]. The cyclo (Arg-Gly-Asp-D-Tyr-Lys) peptide [c (RGDyK)] exhibits high affinity to integrin α_v_β_3_, where its expression on cerebral vascular endothelial cells is induced by ischemia [29, 30]. Administration of c (RGDyK)-conjugated exosomes has resulted in a strong suppression of the inflammatory response and cellular apoptosis in the lesion region after cerebral ischemia [31]. The GE11 peptide has high affinity for the epidermal growth factor receptor and GE11-positive exosomes that contained the miRNA let-7 were able to prevent tumor growth [32]. In addition, there are other proteins which would be able to dock exosomes to certain cells, such as connexin 43 [33], connexin 26 [34] and tenascin C [35].

Exosomes are naturally occurring nanosized vesicles secreted by a variety of cells [36]. The exosomes play important roles in long-distance intercellular communications facilitating transfer of microRNAs, functional mRNAs and proteins [37]. The exosomes can avoid phagocytosis by macrophages due to the small size and be naturally stable to escape endosome/lysosome dependent degradation compared with liposomes and polymeric nanoparticles. Most importantly, accumulating evidences showed that exosomes could cross BBB which has been proven to be the major obstacle for delivery of drugs to the CNS. Recent findings revealed that communication mediated via exosomes is a major mediator of physiological and pathological processes in the CNS [38]. Compared with other kinds of cells, cultured MSC could produce exosomes in a larger scale [39]. It has been suggested that MSC-derived exosomes possess enzymatically active NEP and decreased both extracellular and intracellular Aβ levels in N2a cells [7]. Indeed, intravenously injection of MSC derived exosomes sharply improved learning and memory of AD mice according to our previous work [8] and this study. However, as NSCs have been proven to have great potential to treat AD [3], it would be very interesting to compare the effects of exosomes from MSCs or NSCs on the improvement of learning and memory. At present, questions regarding the isolation and production processes, quality and quantity of exosomes impact the potency of exosome-based strategy remain unanswered. Besides, the dosage of exosome administered was from 10^9^ to 10^12^ according to the literatures and 5 × 10^11^ exosomes were adopted in this study. The quantity and dosage of exosomes for the treatment of AD through intravenous injection should be further optimized. Despite these challenges, the emerging field of exosome-based therapy offers many opportunities for the treatment of AD.

Inflammatory response has long been implicated in the process of neurodegeneration seen in AD sufferers [40]. TNF-α, IL-1β and IL-6, as pro-inflammatory cytokines, showed a significant increase in AD mice and the levels of these cytokines may be directly related to the amount of soluble and insoluble Aβ present in the brain [41]. The pleiotropic cytokines IL-4, IL-10 and IL-13, as anti-inflammatory factors, inhibit the synthesis and release of pro-inflammatory cytokines. IL-10 was found to suppress Aβ and lipopolysaccharide-induced inflammatory proteins including TNF-α, IL-1β and IL-6, while IL-4 and IL-13 reduced IL-6 secretion [42]. IL-4 also could attenuate the neuroinflammation induced by amyloid-beta in vivo and in vitro [43]. The nanomaterial, graphene quantum dots (GQDs) conjugated neuroprotective peptide glycine-proline-glutamate improved learning and memory capability, downregulated pro-inflammatory cytokines (TNF-α, IL-1β and IL-6), and upregulated the anti-inflammatory cytokines (IL-4 and IL-10) in APP/PS1 transgenic mice [44]. The data presented in this study found the changes of anti-inflammatory factors were not obvious in the mice intravenously injected with unmodified exosomes from MSC. However, administration of MSC-RVG-Exo not only decreased the level of TNF-α, IL-1β and IL-6, but also increased the level of IL-4, IL-10 and IL-13 in APP/PS1 mice. APP/PS1 mice began to exhibit elevated expression of GFAP from at least 7 months [45]. We also identified that injection of MSC-Exo and MSC-RVG-Exo could significantly attenuate the expression of GFAP. Moreover, the AD mice treated with MSC-RVG-Exo showed the lowest expression of GFAP. Upregulation of GFAP has been reported in animal models of AD, which suggests that astrocytes are believed to play a central role in the pathogenesis of AD [46]. Astrocytes are involved in the development of AD through upregulating the expression of pro-inflammatory cytokines and chemokines as well as regulating the generation and degradation of Aβ [47]. However, further investigations will reveal molecular mechanisms underlying the anti-inflammatory properties of exosomes derived from MSCs.

## Conclusions

In summary, our current study demonstrated that the CNS-specific peptide RVG modified MSC-exosomes improved learning and memory function in APP/PS1 transgenic mice. In addition, our results showed that RVG modified MSC-exosomes could effectively reduce Aβ accumulation and activation of glial cells, and balanced the inflammatory factors in the brain of AD mice. Thus, brain targeted exosomes derived from MSCs may rescue memory deficits by regulating inflammatory responses and well serve as a next generation drug for the treatment of AD and potentially for other neurodegenerative diseases.

## Additional file

Additional file 1: **Figure S1**. The characteristics of MSCs was detemined by immunocytofluorescense. Most of the cells were postive for CD29 and CD44. Scale bar = 25 μm. (TIF 1360 kb)

## Abbreviations

AD: Alzheimer’s disease
Aβ: amyloid-β peptide
BBB: blood-brain barrier
CHP: cardiac homing peptide
CNS: central nervous system
DMEM: Dulbecco’s modified eagle’s medium
FBS: fetal bovine serum
MOR: opioid receptor Mu
MSC-Exo: exosomes derived from MSCs
MSC-RVG-Exo: RVG-conjugated MSC-Exo
MSCs: mesenchymal stem cells
NSCs: neural stem cells
PBS: phosphate buffer solution
RVG: rabies viral glycoprotein
