# Supplementary figures and images for: RVG-modified exosomes derived from mesenchymal stem cells rescue memory deficits by regulating inflammatory responses in a mouse model of Alzheimer’s disease
Source: Immun Ageing. 2019 May 13;16:10. doi: 10.1186/s12979-019-0150-2 (PMC6515654; doi:10.1186/s12979-019-0150-2)

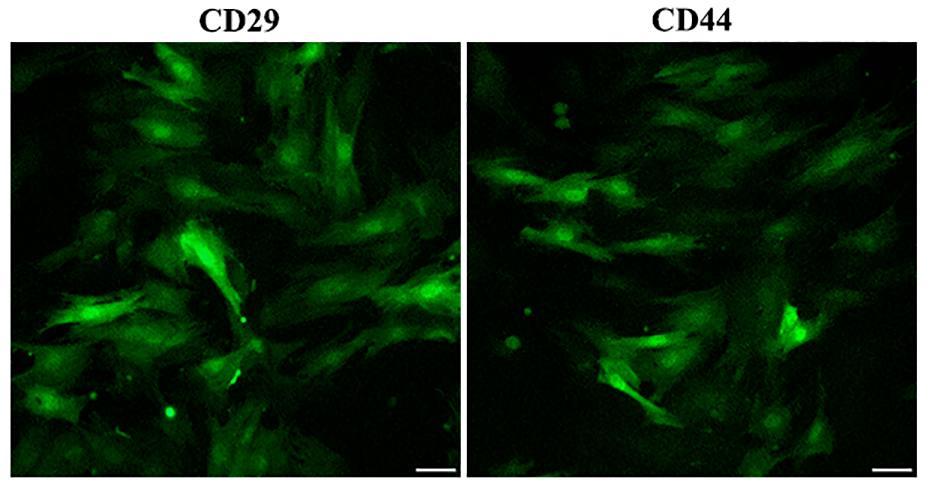

Supplement: Supplementary file 1 — Figure S1. The characteristics of MSCs was detemined by immunocytofluorescense. Most of the cells were postive for CD29 and CD44. Scale bar = 25 μm. (TIF 1360 kb) [file 12979_2019_150_MOESM1_ESM.tif]
